# A transcriptome map of perennial ryegrass (*Lolium perenne* L.)

**DOI:** 10.1186/1471-2164-13-140

**Published:** 2012-04-18

**Authors:** Bruno Studer, Stephen Byrne, Rasmus O Nielsen, Frank Panitz, Christian Bendixen, Md Shofiqul Islam, Matthias Pfeifer, Thomas Lübberstedt, Torben Asp

**Affiliations:** 1 Department of Molecular Biology and Genetics, Faculty of Science and Technology, Research Centre Flakkebjerg, Aarhus University, Forsøgsvej 1, 4200, Slagelse, Denmark; 2 Department of Molecular Biology and Genetics, Faculty of Science and Technology, Research Centre Foulum, Aarhus University, Blichers Allé 20, 8830, Tjele, Denmark; 3 Institute of Bioinformatics and Systems Biology, Helmholtz Center Munich, German Research Center for Environmental Health, Ingolstaedter Landstrasse 1, 85764, Neuherberg, Germany; 4 Department of Agronomy, Iowa State University, 1204 Agronomy Hall, 50011, Ames, IA, USA

**Keywords:** Illumina GoldenGate genotyping, *In silico* SNP discovery, Next generation sequencing (NGS), Perennial ryegrass (*Lolium perenne* L.), Single nucleotide polymorphism (SNP), Transcriptome sequencing

## Abstract

**Background:**

Single nucleotide polymorphisms (SNPs) are increasingly becoming the DNA marker system of choice due to their prevalence in the genome and their ability to be used in highly multiplexed genotyping assays. Although needed in high numbers for genome-wide marker profiles and genomics-assisted breeding, a surprisingly low number of validated SNPs are currently available for perennial ryegrass.

**Results:**

A perennial ryegrass unigene set representing 9,399 genes was used as a reference for the assembly of 802,156 high quality reads generated by 454 transcriptome sequencing and for *in silico* SNP discovery. Out of more than 15,433 SNPs in 1,778 unigenes fulfilling highly stringent assembly and detection parameters, a total of 768 SNP markers were selected for GoldenGate genotyping in 184 individuals of the perennial ryegrass mapping population VrnA, a population being previously evaluated for important agronomic traits. A total of 592 (77%) of the SNPs tested were successfully called with a cluster separation above 0.9. Of these, 509 (86%) genic SNP markers segregated in the VrnA mapping population, out of which 495 were assigned to map positions. The genetic linkage map presented here comprises a total of 838 DNA markers (767 gene-derived markers) and spans 750 centi Mogan (cM) with an average marker interval distance of less than 0.9 cM. Moreover, it locates 732 expressed genes involved in a broad range of molecular functions of different biological processes in the perennial ryegrass genome.

**Conclusions:**

Here, we present an efficient approach of using next generation sequencing (NGS) data for SNP discovery, and the successful design of a 768-plex Illumina GoldenGate genotyping assay in a complex genome. The ryegrass SNPs along with the corresponding transcribed sequences represent a milestone in the establishment of genetic and genomics resources available for this species and constitute a further step towards molecular breeding strategies. Moreover, the high density genetic linkage map predominantly based on gene-associated DNA markers provides an important tool for the assignment of candidate genes to quantitative trait loci (QTL), functional genomics and the integration of genetic and physical maps in perennial ryegrass, one of the most important temperate grassland species.

## Background

High density genetic linkage maps are important tools for QTL fine mapping, map-based cloning, comparative genome analysis and the integration of genetic and physical maps. Several genetic linkage maps based on various markers technologies are now available for perennial ryegrass [1-9]. These maps of moderate marker densities have proved valuable for mapping QTL to broad genome regions. Public marker resources recently established provide the opportunity to increase marker density of these maps, thereby improving map resolution [10-13].

For example, the genetic linkage map of the perennial ryegrass mapping population VrnA has initially been used for a QTL study to characterise vernalization response and contained 93 markers spanning 490.4 cM with an average distance between markers of 5 cM [2]. This map has been complemented over time with candidate gene-based CAPS markers to study disease resistance traits [14,15] and contained around 180 markers with total map length of 487 cM when used to evaluate seed yield and fertility traits [16]. Recently, the same map has been used to localise genes involved in water stress and contained 222 markers, between 24 and 37 on each linkage group (LG), spanning a total of 736 cM [17].

Among the different marker technologies available to increase the density of a genetic linkage map, SNPs have attracted much interest, mainly for two reasons: Firstly, SNPs are the most abundant form of genetic variation [18] and occur at regular intervals in the genome [19]. Secondly, SNPs are highly suitable for multiplexed genotyping assays on mass spectrometry, microarray or beadarray-based platforms [20]. Advancements in these technologies has enabled increased throughput at low cost per data point.

The potential of SNPs for extensive genome analysis has been impressively demonstrated in model plant species such as *Arabidopsis thaliana*, rice (*Oryza sativa*), and maize (*Zea mays*), where fully sequenced genomes resulted in the identification of millions of SNPs suitable for genome-wide association studies and molecular breeding concepts such as genomic selection [21].

In species where a reference genome sequence has not been established yet, several strategies for large-scale SNP discovery have been reported, mainly being divided into in vitro and *in silico* approaches. Amplicon resequencing is an in vitro approach and has proven very reliable for SNP identification with a false discovery rate usually below 5% [22]. Furthermore, cloned PCR fragments and allele-specific sequencing allow haplotype identification at sufficient read lengths and the discrimination of orthologous (allelic) and paralogous (derived from closely related genes or highly conserved domains in gene families) sequences. However, amplicon resequencing requires an enormous effort for large scale studies, since each gene needs to be amplified individually and thus might have limited application in the future. Despite the labour intensive nature of amplicon cloning and sequencing, this has been the method of choice for SNP discovery in ryegrasses to date [23]. For *in silico* SNP discovery, the rapidly growing public EST databases can be exploited as a potential sequence resource [24,25]. This approach has been applied in other Poaceae crop species including wheat (*Triticum aestivum* L.) [26] and barley (*Hordeum vulgare* L.) [27]. However, availability and quality of public ryegrass EST sequences are often limited and it might be difficult to obtain a sufficient number of EST reads from the same gene, a key factor for reliable *in silico* SNP identification [22,28]. As a result of these limitations, the percentage of false discovery rates is often considerably high and can vary between 5 and 50% [22]. Recent advances in NGS opened up the opportunity for whole genome resequencing as an extremely powerful strategy for *in silico* SNP discovery at appropriate sequence coverage. However, *de novo* assembly of short NGS reads is difficult in outbreeding species with a highly heterozygous, large and complex genome containing a high degree of repetitive elements. Moreover, whole genome resequencing may not be necessary to target recombination blocks present in bi-parental mapping populations. Therefore, different strategies for complexity reduction such as reduced representation libraries (RRL) have been proposed to sequence only a subset of the genome for SNP discovery [29]. RRLs have been applied in a wide range of plant species such as maize [30], rice [31], grapevine species (*Vitis* spp.) [32], common bean (*Phaseolus vulgaris* L.) [33] and soybean (*Glycine max* L.) [34]. Another strategy for complexity reduction is transcriptome sequencing [35,36], where expressed genes are targeted and highly repetitive non-transcribed genomic regions are excluded. This emerged as an efficient method for the high-throughput acquisition of gene-associated SNPs [37,38].

For SNP genotyping in a scale up to 3,072 SNPs, the Illumina GoldenGate technology [39] has successfully been used in several crop species. In diploid barley, for example, custom oligo pool assays (OPAs) have been designed to estimate linkage disequilibrium (LD) in inbred elite varieties [40] and for genetic linkage mapping [41]. Recently, two validated 1,536-SNP barley OPAs (BOPA1 and BOPA2) were made available to the barley community as an excellent marker resource in terms of distribution and density in the barely genome, technical performance and biological importance [42]. In more complex genomes such as soybean, GoldenGate genotyping has been used for linkage mapping in recombinant inbred line mapping populations [43]. While also being autogamous, soybean contains around twice as many gene paralogues (32%) when compared to 16% in barley [44], which is known to affect the success rate of multiplexed high-throughput genotyping methods [45,46]. However, the rate of 89% successfully scored SNPs indicated that the genome complexity of soybean had limited impact on GoldenGate performance in a carefully selected SNP panel [43]. In maize, the genome contains about 80% repetitive sequences and a similar amount of paralogous sequences as soybean [44], but a substantially higher intraspecific genetic variation [47]. Despite this, OPAs containing 1,536 SNPs designed from publicly available SNPs (http://www.panzea.org) are routinely used for diversity, linkage and association analysis, as well as for LD estimations [48,49]. To date, the GoldenGate assay proved even successful for SNP genotyping in tetraploid and hexaploid wheat lines [50] and allopolyploid *Brassica napus*[51].

Encouraged by this, we developed the first open access Lolium 768-SNP OPA (thereafter referred to as LOPA1) for the allogamous forage grass species *L. perenne* with a genome size and complexity comparable to maize. Specifically, we aimed at (i) developing an efficient strategy for *in silico* SNP discovery based on next generation transcriptome sequencing, (ii) implementing a pipeline for successful OPA design, (iii) getting first insights to cross-species amplification rates of ryegrass SNPs and (iv) constructing a high density EST map in perennial ryegrass as a promising tool for QTL fine mapping, map-based cloning and comparative genome analysis.

## Results

### SNP discovery

A comprehensive EST collection consisting of a total of 31,379 ryegrass ESTs generated by Sanger sequencing was subjected to quality filtering and vector clipping, resulting in 25,744 high-quality EST reads of 8.5 Mbp nucleotide information [52]. A *de novo* assembly using the PHRED, PHRAP, and CROSS_MATCH software packages resulted in 9,399 non-redundant contigs and singletons with an average length of 889 bp, thereafter referred to as unigene set.

For SNP discovery, 454 GS FLX transcriptome sequencing of the parents of VrnA and a ryegrass genotype that has been inbred for six generations was performed. In total, 802,156 high-quality reads with an average read length of 377 bp were aligned against the unigene set. A minimum of four reads at the SNP position and at least two reads for each SNP variant was required for SNP calling. A total of 15,433 SNPs in 1,778 of these unigenes met the stringent SNP calling parameters, out of which one SNP in each unigene was selected for further analysis.

### SNP selection, validation and Lolium oligo pool assays (LOPA1) design

Out of a total of 1,778 SNP-containing unigenes, 556 (31%) were discarded because (i) the detected SNPs were located within a distance of 30 bp to the sequence end or intron/exon splice junctions estimated by BLASTN analysis against the rice genome sequence, (ii) additional SNPs and/or InDels were observed within a distance of 30 bp to the target SNP, or (iii) the reference inbred genotype revealed allelic sequence polymorphisms, indicating the presence of similar but non-allelic sequences in the alignment. For another 132 unigenes (7%), no significant (E < e-10) sequence similarities to the rice genome sequence were found by BLASTN analysis, making a proper positional prediction of intron/exon splice junctions impossible. Moreover, sequence reads from only one parental genotype were observed for 72 (4%) of the SNP-containing unigenes.

In order to validate the remaining 1,018 SNPs prior to the GoldenGate assay, a subset of 22 randomly selected SNPs were tested either by direct sequencing of PCR fragments amplified from the mapping parent(s) being polymorphic for the respective SNP or by high resolution melting (HRM) curve analysis of short amplicons covering the predicted SNP polymorphism (Additional file 1: Figure S1A and S1B). As a result, 17 (77%) out of the 22 examined SNP candidates were experimentally confirmed and represented biological SNPs. Sequencing failed for two SNPs and an additional three (14%) were monomorphic. These five SNPs were excluded from further analysis.

The remaining 1,013 SNPs were subjected to functionality score calculation by Illumina Technical Service, out of which 253 (13%) yielded scores lower than 0.6 and were, therefore, discarded. For eight out of 760 unigenes, two SNP markers were selected for genotyping. Finally, 768 SNPs satisfying the stringent selection criteria were used to design the 768-plex LOPA1.

### GoldenGate genotyping and allele calling

**Figure 1:**
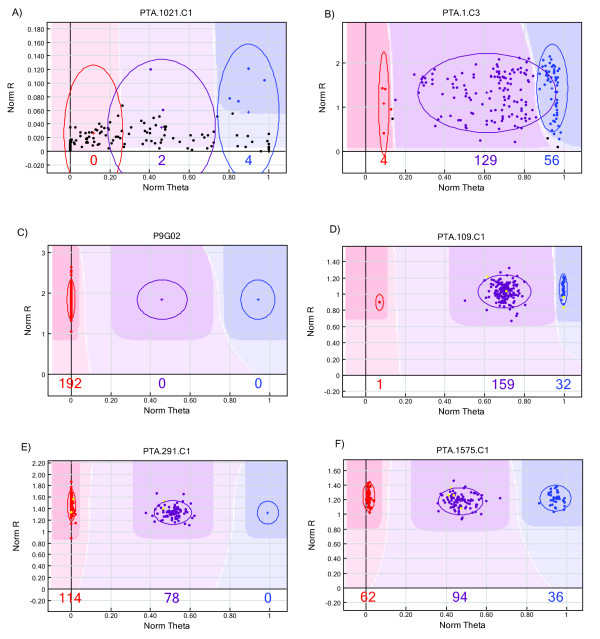
**Examples of SNP graphs observed in Lolium oligo pool assay (LOPA1) GoldenGate genotyping.** SNP graphs are illustrated using the Software Illumina® GenomeStudio, version 2009.2. The normalized R (y-axis) is the normalized sum of intensities of the two dyes (Cy3 and Cy5), the normalized Theta (x-axis) is the deviation of Cy3 and Cy5 fluorescence from pure Cy3 and pure Cy5 signal (0 and 1). A normalized Theta value close to 0 and 1 is homozygous for SNP variant 1 and 2, respectively, a heterozygous sample is in between. The red, blue and purple ovals have the diameter of two standard deviations computed from the dispersal of the red, blue and purple dots, respectively. The numbers of plants in each cluster are indicated below the x-axis. (**A**) The 192 samples genotyped for SNP marker PTA.1021.C1 revealed fluorescence signal intensities close to 0, indicating assay failure. (**B**) Although the clustering algorithm at SNP PTA.1.C3 distinguished the three clusters at a GenTrain score of 0.40, such a genotyping pattern was considered inaccurate and this SNP was discarded from further analysis. (**C**) This illustration shows the SNP graph of monomorphic P9G02. (**D**) and (**E**) illustrate dominant SNPs being homozygous in one and heterozygous in the other mapping parent. For genetic linkage mapping, the markers PTA.109.C1 and PTA.291.C1 followed the segregation type nnxnp and lmxll, respectively [53]. Dots corresponding to the parents of the VrnA mapping population (which are represented in duplicates) are highlighted in yellow. Graph (**F**) shows a classical example of a SNP marker being heterozygous in both parents following the segregation pattern hkxhk.

The GoldenGate assay failed for 76 out of 768 genotyped SNPs (10%) and poor or inaccurate fluorescent signals were detected (see Figure 1A as an example). Of the remaining 692 SNPs, 100 (14%) did not form clusters reliably separating genotypes and/or revealed cluster separation scores lower than 0.8 (Figure B1). Additional 83 SNPs (12%) were monomorphic in the mapping population (Figure 1C). The remaining 509 SNPs (77%) were segregating either in one (Figure 1D and 1E) or in both mapping parents (Figure 1F) and were available for genetic linkage mapping.

The two duplicated parental genotypes of the VrnA mapping population revealed highly consistent calls. For successfully genotyped SNPs, the frequency of missing values (MV) was below 0.3% within the mapping population.

### Genetic linkage map

**Figure 2:**
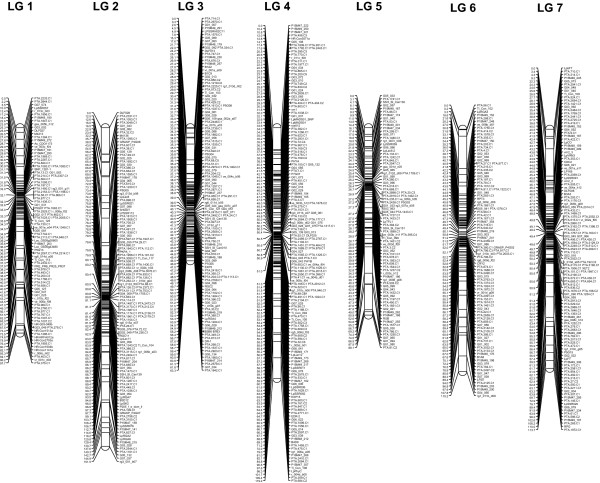
**Transcriptome map of perennial ryegrass (*Lolium perenne*L.).** The EST-based SNPs developed in this study were used to map 495 ryegrass unigenes in the VrnA mapping population using the Haldane mapping function of JoinMap version 4.0 [55]. Linkage groups (LG) were numbered according to the nomenclature accepted for Triticeae, scale units are given in centi Morgan (cM). The resulting VrnA transcriptome map contained 838 DNA markers, ranging from 87 on LG 5 to 168 on LG 4 with an average of 120 markers per LG. Out of these, 767 are EST-derived SSRs, SNPs or CAPS marker. The total map length was 750 cM, spanning from 63 cM on LG3 to 151 cM on LG 2 (mean LG length of 107 cM). The average marker distance was less than 0.9 cM.

The mapping data of the VrnA map described in Jonavičienė et al. [17] and the 509 unigene SNPs were combined and grouped based on independence LOD scores. Markers were assigned to LGs at a LOD ratio threshold of 4.0 with the exception of LG1 and LG3, for which a LOD ratio threshold of 12 was necessary to separate the two LGs from each other. Fourteen SNPs failed to group with existing markers and were, therefore, excluded from mapping. Thus, a total of 495 SNP loci associated with transcribed genes (64% of the SNPs selected for GoldenGate genotyping) were located on the genetic linkage map (Additional file 2). The resulting VrnA map contained 838 DNA markers, ranging from 87 on LG 5 to 168 on LG 4 with an average of 120 markers per LG, of which a total of 767 are gene-derived SSRs, SNPs or CAPS markers (Figure 2). Markers were clustered around centromeric regions (Figure 2, Additional file 3: Figure S2). In order to estimate the accuracy of marker positions, 6 unigenes (PTA.1007.C, PTA.126.C1, PTA.404.C2 PTA.169.C3 PTA.609.C3 PTA.796.C3) were mapped based on more than one SNP. All SNPs derived from the same unigene mapped with a distance of 1.5 cM, four of them within less than 0.2 cM. Similarly, two CAPS and a SNP marker derived from the *LpVrn1* gene mapped within less than 1.9 cM, whereas a CAPS and a SNP marker for *LpCO* mapped within the same cM. Another set of 18 SNPs were derived from unigenes previously mapped by EST-SSRs [54], allowing to compare performance and accuracy of SSR and SNP markers for genetic linkage mapping. Of the 18 comparisons, 11 (61%) mapped within 0.5 cM and only three that were located at the telomeric ends of the LGs, differed more than 3 cM. The slightly higher discrepancy of SNP and SSR map positions was an effect of the higher MV rate observed during SSR genotyping (data not shown).

**Figure 3:**
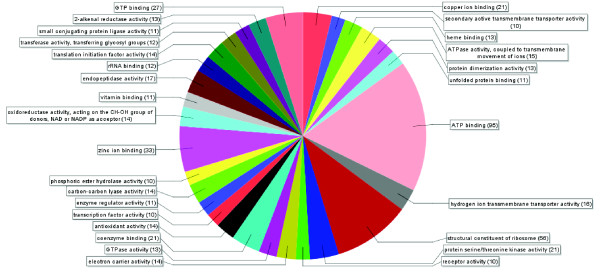
**Description of the molecular functions of mapped Lolium unigenes.** Mapped unigenes were grouped into functional classes based on Gene Ontology (GO) using the Blast2GO search tool [56] and represented a broad spectrum of molecular functions active in different cellular components.

Of the 732 non redundant expressed genes mapped in VrnA, 654 (89%) revealed significant (E < e-10) sequence similarities in a BLASTX search against the non-redundant (nr) protein database of GenBank, out of which 600 (82%) corresponded to genes with known molecular functions active in different cell components (Figure 3, Additional file 4: Figure S3, Additional file 5: Figure S4, Additional file 6: Figure S5). Unigenes were grouped in functional classes representing binding and catalytic activities (42% and 36%, respectively), structural molecule activities (8%), transport activities (7%), molecular transducer and transcription activities (2% each), enzyme regulatory activities (1%), as well as genes involved in nutrient uptake and transport (<1%).

The total map length was 750 cM, ranging from 63 cM on LG3 to 151 cM on LG 2 (mean LG length of 107 cM) with an average marker distance of less than 0.9 cM (Figure 2).

### Intra- and interspecific cross amplification

In addition to the VrnA mapping population including parental and grandparental genotypes, eight parental plants of four different perennial ryegrass mapping populations, one parent of the p150/112 intraspecific ILGI reference population [4] and the two parental genotypes of the Italian ryegrass (*Lolium multiflorum* Lam.) mapping population Xtg-ART [57] were used for genotyping. This allowed an estimation of the transferability of these SNPs to other genetic backgrounds. Of the 592 successfully genotyped SNPs, 275 (47%) detected reliable polymorphisms in at least one of the four additional perennial ryegrass mapping populations (between 201 and 250 for each population, Table 1), 48 of them (8%) were segregating in all populations. A total of 131 SNP markers (17%) detected polymorphisms segregating in Xtg-ART (Table 1). Interestingly, marker PTA.1032.C1 failed GoldenGate genotyping for perennial, but produced clear calls for the two Italian ryegrass plants. Markers PTA.32.CB2, PTA.43.C1, PTA.103.C1, PTA.271.C2, PTA.1535.C1, PTA.1613.C1, PTA.2333.C1, PTA.2371.C1 and r_005b_a08 were monomorphic in perennial ryegrass with a distinct genotype in Italian ryegrass. PTA.240.C2 and PTA.1044.C1 were monomorphic in perennial ryegrass but segregated in the Italian ryegrass mapping population Xtg-ART.

**Table 1:** Intra- and interspecific cross amplification rates of SNPs on the Lolium oligo pool assay (LOPA1)

**Species**	**Mapping population**	Average call rate of all SNPs*	**Number (percentage) of SNPs generating signals**	**Number (percentage) of polymorphic SNPs referred to SNPs generating signals**	**Number (percentage) of polymorphic SNPs referred to 768 SNPs on LOPA1**
*Lolium perenne* L.	VrnA	0.914 a	692 (90%)	509 (74%)	509 (66%)
	DLF1	0.846 b	567 (74%)	241 (43%)	241 (31%)
	DLF2	0.858 b	605 (79%)	235 (39%)	235 (31%)
	DLF3	0.840 b	598 (78%)	201 (34%)	201 (26%)
	DLF4	0.850 b	601 (78%)	250 (42%)	250 (33%)
	ILGI	0.882 c	665 (87%)	†192 (29%)	192 (25%)†
*Lolium multiflorum* Lam.	Xtg-ART	0.818 d	557 (73%)	131 (24%)	131 (17%)

*Mapping populations with different letters vary significantly at *P* < =0.05 in their average call rates.

†Estimation from one parent only.

SNP performance of LOPA1 in VrnA was compared to different *Lolium multiflorum* Lam. (Xtg-ART) and *Lolium perenne* L. (DLF1 to 4) mapping populations, each represented with the two parental genotypes. For each mapping background, the average call rate of all SNPs is given and varies significantly at *P* < =0.05 if indicated with different letters. SNPs with a clear fluorescent signal detected in both mapping patents are considered as “SNPs generating signals”. The numbers and percentages of polymorphic SNPs refer to SNP markers being heterozygous either in one or both parents, indicating a segregation pattern of lmxll, nnxnp or hkxhk [53], respectively, in the corresponding mapping population. For the ILGI reference population represented with one parental genotype only, the number of SNPs being heterozygous is given. The percentages of polymorphic SNPs are refereeing to the number of SNPs generating signals and the total number of SNPs on LOPA1.

## Discussion

In recent years, technological advances in methods for high-throughput detection and genotyping of SNP markers have initiated a novel era in using molecular markers for genome analysis and breeding applications [58]. But still, the use of SNP markers for large-scale genome studies in allogamous forage grass species such as perennial ryegrass is still in its infancy. This is due to the low number of publicly available SNPs and the challenge of efficient SNP discovery and genotyping in a highly heterozygous genome containing a high proportion of repetitive elements and paralogous sequences. Here, we present both; an efficient SNP discovery pipeline based on 454 GS FLX transcriptome sequencing, and an Illumina GoldenGate assay to genotype, validate, and map the identified SNPs in the two way pseudo-testcross population VrnA.

### Genic SNP discovery in complex genomes

Transcriptome resequencing strategies and subsequent *in silico* SNP discovery have emerged as an efficient strategy for large-scale SNP discovery [29,37,58-63]. However, time and cost benefits are counterbalanced by a higher false discovery rate compared to *in vitro* approaches [64,65]. Incorrectly detected SNPs are primarily due to paralogous gene sequences interfering with the assembly of short NGS reads. In the present study, this was resolved by using a ryegrass unigene set with an average length of 889 bp as a reference for the assembly of the shorter 454 GS FLX transciptome reads. The power of such an approach to separate paralogous sequence variation has recently been shown in salmonids, whose genome contains a high degree of paralogous sequences due to a recent whole genome duplication event [66]. Moreover, a highly inbred ryegrass genotype was included for transcriptome sequencing as a means to identify paralogous genes and sequences from highly conserved domains of gene families in the alignment. As the inbred genotype was self-pollinated for six generations, the overall degree of heterozygosity is less than 1.5%. Genes that showed polymorphisms in reads from the inbred genotype indicated the presence of similar, non-allelic sequences and were therefore discarded for SNP discovery, thereby providing a reliable tool not only to reduce false positives in SNP discovery but also to facilitate the identification of genotype clusters during SNP genotyping.

Sequencing errors may represent an additional source of false positive SNPs. Even though error rates of NGS platforms are low (usually less than 1%) [67], a combination of Sanger sequencing (used for the establishment of the unigene set) and NGS (for transcriptome deep sequencing) was applied. Error rates of such combined sequencing approaches are even lower and thus an insignificant source of false-positive SNPs [68]. As a result, the present study revealed a false discovery rate (i.e., monomorphic SNP rate) of less than 12%, even lower than the initial estimation of 14%. The proportion of successfully called to finally mapped SNPs of 72% is comparable or slightly higher to validation rates between 57% and 77% observed in other species such as *Brachypodium distachyon*[69] or rye (*Secale cereale* L.) [63]. In conclusion, sequencing depth and a proper handling of paralogous sequences go hand in hand and are key factors for successful *in silico* SNP discovery approaches based on RNA-seq. In future, large scale NGS achieving longer read lengths and higher throughput in combination with improved assembly algorithms will provide opportunities for similar *in silico* SNP discovery approaches in less characterized species.

### Lolium oligo pool assays (LOPA1) design for ryegrass SNP genotyping

Highly multiplexed Illumina SNP arrays are efficient tools to enhance mapping of expressed genes, thereby improving the resolution and usefulness of a genetic linkage map [42,48,69-73]. The use of a community OPA containing validated and well-performing SNPs as available for barley [42] is straightforward. However, the high calling rate (the rate of successfully genotyped SNPs) is often compromised by a lower conversion rate (the rate of polymorphic SNPs), as these SNPs were not a priori screened for polymorphisms within a particular mapping population. This was observed in barley, where approximately 51% of SNPs in the BOPA1 were polymorphic in a barley doubled haploid (DH) population [41]. Similarly, high calling (90%) but limited conversion rates (39 to 53%) were obtained when *de novo* OPA design was based on validated SNPs selected from public databases [48]. The percentage of polymorphic SNPs was even lower in *Pinus* and *Picea* species and ranged between 12 to 19% [65], which might be an effect of the very large and complex genomes [74], as well as limited sequence resources established for these species.

In contrast, much higher rates of polymorphic SNPs can be achieved by transcriptome resequencing of parental genotypes in the target mapping population, allowing the design of customized OPAs containing SNPs that are segregating in the mapping pedigree. While this was very efficient to generate informative SNPs for linkage mapping, it might compromise the transferability of these SNPs to different genetic backgrounds. Given the high impact of additional polymorphisms in the flanking sequence of the target SNP on genotyping performance [75], intra- and interspecific SNP amplification rates in ryegrass might per se be lower when compared to inbreeding species due to increased nucleotide diversity present in outbreeding species. The detected 15,433 SNPs in 1,778 unigenes (this is an average of nine SNPs per unigene, one SNP every 102 bp) reflected the high nucleotide diversity present in a set of only four haplotypes. Nevertheless, the percentage of SNPs generating clear fluorescent signals (73 to 87%) was high in other Italian and perennial ryegrass backgrounds. Estimated rates of polymorphic SNPs ranging up to 33% indicate that LOPA1 can be applied to different genetic backgrounds. However, a more detailed study based on larger collections of various ryegrass genotypes will be required to confirm the significance of the reported SNP markers for broad-scale applications in ryegrasses. With the aim to further improve our *in silico* SNP discovery pipeline, the 76 SNPs failing GoldenGate genotyping were further examined and mapped back to genomic DNA. Interestingly, over 90% of these 76 SNPs had exon-intron boundaries within 20 bp flanking the target SNP (data not shown). This highlights an important drawback when developing SNPs from transcriptome sequencing data and indicates that BLASTN analysis to the rice genome sequence was inefficient to identify introns in ESTs for about 10% of the unigenes. A reference genome sequence will prove very useful to exactly locate intron-exon junctions for future large-scale SNP discovery studies.

### Implications of the transcriptome map for ryegrass genetics and genomics

The ryegrass transcriptome map displays the genetic location of 732 expressed genes putatively underlying specific biochemical or physiological functions that control variation for agronomically important traits. The VrnA population has already proven to be valuable for mapping and cloning of major genes associated with meristem identity and the control of floral transition such as *LpVrn1LpCO,* and *LpVrn3*[76,77]. For the same traits, the present transcriptome map contains additional candidate genes such as the TERMINAL FLOWER1-like gene (*LpTFL1*) that is a well characterised repressor of flowering and a controller of axillary meristem identity in ryegrass [78], and a homologue of the *Triticum monococcum* L. gene *TmVIL3,* that is up-regulated by vernalization [79]. The Arabidopsis homoloque of *VIL3* is known to mediate chromatin modifications for stable repression of the *FLOWERING LOCUS C* (*FLC*). Interestingly, the ryegrass homologue of *TmVIL3* (ve_003c_f04) mapped close to the centromere on LG1, syntenic to the map position of *TmVIL3* in *T. monococcum*.

Another key trait that relates to vernalization response is fructan content, and the accumulation of fructans during cold acclimation. Fructans are known to play a key role in crop plants in response to abiotic stress in general, including drought, cold and freezing tolerance in particular [80]. In the present study, previously characterised, as well as novel genes involved in fructan biosynthesis were mapped, providing the opportunity to study fructan related metabolic processes involved in abiotic stress tolerance of grasses. This might be of particular interest since the VrnA grandparents – originating from different geographical latitudes – are not only significantly contrasting for their vernalization requirement, but also for the ability to accumulate fructans during cold acclimation, as well as in the response to drought treatment (unpublished data). Thus, given the high degree of segregation for traits such as abiotic stress tolerance and fructan accumulation in the VrnA population, it does represent a unique tool to unravel the gene regulatory networks of these traits.

Similarly, the current map contains genes involved in resistance to various biotic agents. Apart from the previously published NBS-LRR homologues [14,15], the map locates elements from disease resistance signal transduction pathways (Pto kinase interactor 1, p_001c_b08 corresponding to G02_079) that were shown to be up-regulated after *Xanthomonas translucens* pv. *graminis* (Xtg) infection causing bacterial wilt [81]. Another gene showed high sequence similarity to members of the family of germin-like proteins (GLP; r_010d_c02) that are known to be involved in broad-spectrum basal defence against various pathogens and are also induced upon abiotic stress [82].

Other research groups can take advantage of this resource by using the unigene sequence information to develop simple ‘Blind Mapping’ HRM assays [77] to map a well distributed subset of the markers in their favourite mapping populations. This can then aid the transfer of information between different populations and species. The transcriptome map also serves as a source of candidate genes involved in various biological processes and molecular functions for association mapping. With an average marker distance of less than 0.9 cM, the presented VrnA map represents a good starting point for the establishment of BAC contigs for any genomic region of interest and will, in combination with the in-house BAC library established from one VrnA parental genotype [83], provide a very efficient toolbox for map-based cloning and gene isolation. However, it is worth noting that markers were not evenly distributed along the LGs, but clustered around the centromeres. Clustering of genes towards genetic centromeres due to low recombination frequencies is well known and has been described in barley [84,85] and Brachypodium [69]. As a consequence, some markers at the centromeres could not be separated by 184 mapping individuals and co-segregated within recombination blocks. Thus, effects of MV in mapping data became more apparent and single MV resulted in slight changes of map positions, thereby explaining mapping discrepancies of two markers derived from the same unigene. We conclude that the current linkage map comes close to saturation of markers, at least in centromeric regions, and rather more mapping individuals than more markers would further improve map resolution. However, besides the general tendency that recombination frequency is reduced at genetic centromeres, it can vary dramatically along the chromosome [69]. *In silico* mapping of the unigene sequences to the ryegrass genome sequence, when available, will help resolve to what extent recombination frequencies vary along the chromosomes in greater detail, and will be valuable for ordering and orientation of scaffolds into pseudomolecules during the assembly of a ryegrass reference genome.

The availability of fully sequenced model grass genomes such as rice, Brachypodium, maize, and sorghum (*Sorghum bicolor* L. Moench) enables efficient exploitation of grass genome sequence resources for genetic and breeding applications in ryegrasses. Once established, syntenic relationships allow transferring map and marker information from related species across conserved genome regions [86]. Early comparative studies between the Pooideae tribes Triticeae and Poeae relied on restriction fragment length polymorphism (RFLP) markers mapped across different species and found that the genetic maps of perennial ryegrass and the Triticeae cereals are highly conserved in terms of orthology and colinearity [87,88]. However, these results were obtained from low-resolution genetic maps containing a limited number of anchor RFLP markers that allowed the detection of large rearrangements only, thereby missing a substantial part of the existing micro-synteny. Map and sequence-based markers presented here provide the opportunity to update and redefine synteny between ryegrass and the fully sequenced model grass genomes at a higher level of resolution to address micro-colinearity structure.

### Future prospective of high throughput SNP discovery and genotyping

The advancements in sequencing and genotyping technology were a prerequisite for the work described here, and further improvements in throughput of NGS instruments can be expected. Combined with decreasing costs, it is worth considering genotyping by sequencing (GBS) approaches, thus by-passing the necessity for array-based genotyping [89]. In this case, we move straight to genotyping by means of sequencing all individuals of a mapping or association panel. GBS strategies will prove extremely powerful for genome-wide association studies and for plant breeders moving towards implementing genomic selection in their breeding programmes [90].

However, whole genome resequencing may not be necessary when working within bi-parental mapping populations, where – depending on the population size – a finite amount of recombination and genome reshuffling is present. Thus, only SNP numbers adequate to cover the recombination blocks in the population are required. In this case, it may be sufficient to sequence a well distributed portion of the genome in all individuals [29]. A cost-effective approach of genotyping by sequencing on a small portion of the genome has recently been described and demonstrated in both maize and barley mapping populations [91]. The method described the use of a simple bar-coding strategy that allowed a high-level of multiplexing (up to 96-plex) and enabled mapping of approximately 200,000 and 25,000 sequence tags in maize and barley, respectively. With the increasing throughput of NGS, the authors envisage multiplexing up to 384 samples per lane, and thus pushing genotyping to under $20 per sample. Although a reference genome is not necessarily required for this approach, it does allow for the use of genotype imputation methods when coverage is low.

Armed with these new powerful genotyping tools we can begin to reconsider how we construct mapping populations in order to improve power and precision. It will now be possible to densely genotype much larger populations for both bi-parental and association mapping studies, with the need for quality phenotyping remaining the sole bottleneck.

## Conclusions

This study demonstrates the efficiency of using next generation transcriptome sequencing to discover gene-associated SNPs in species where no reference genome sequence has been established yet. In addition, we describe a workflow on how to successfully use the Illumina GoldenGate technology in outbreeding species characterized by highly heterozygous, large and complex genomes. We have also demonstrated the transferability of these SNPs to other perennial and Italian ryegrass mapping populations. The resulting map locates candidate genes for agronomically important traits and – at the given map resolution – represents a promising starting point for QTL fine mapping, LD-based association mapping, and map-based cloning via BAC clone isolation and sequencing. Moreover, the present EST map provides new anchor points for detailed studies of comparative grass genomics that will prove useful for future ordering and orientation of scaffolds into pseudomolecules during the assembly of a ryegrass reference genome.

## Methods

### Mapping population

The VrnA two-way pseudo-testcross mapping population consisting of 184 F2 perennial ryegrass genotypes [2] was used to map the EST-derived SNPs. These plants were complemented with eight parental genotypes of four different perennial ryegrass mapping populations, one parent of the p150/112 intraspecific ILGI reference population [4], and two Italian ryegrass plants which have been used to establish the Xtg-ART population characterized for bacterial wilt and crown rust resistance [57,92]. Genomic DNA was isolated from young leaves following a phenol/chloroform extraction protocol with minor modifications described in Jensen et al. [2].

### RNA isolation

Total RNA from both parents of the VrnA population (NV#20 F1-30 and NV#20 F1-39, respectively) as well as the inbred genotype p226/179/2 was isolated using Tri® Reagent (Sigma-Aldrich, St. Louis, MO, USA) according to the manufacturer's instructions. Isolation of mRNA and synthesis of cDNA was performed according to Milano et al. [38].

### SNP discovery

The unigene set was generated according to Asp et al. [52] using the PHRED, PHRAP and CROSS_MATCH software packages [93-95]. For the final assembly, the PHRAP minmatch threshold was 75, all other parameters were set to default. The Roche FLX 454 technology was used to generate reads using barcoded libraries [96] from NV#20 F1-30, NV#20 F1-39 and the inbred genotype p226/179/2. The alignment of the 454 reads to the unigene set was based on the Mosaik sequence assembler (http://bioinformatics.bc.edu/marthlab/Mosaik/). A hash size of 15 was used with a mismatch threshold set to a maximum of 4% mismatches. Large-scale SNP detection in the assembled contigs was performed using GigaBayes V0.4.1 [97] with a minimum of four total reads at each SNP position and a minimum read coverage of two for each SNP variant. Minimum base quality was 10, the probability threshold of each SNP at least 0.5.

### SNP validation

Prior to GoldenGate assay design, a subset of detected SNPs were validated by HRM or direct sequencing of PCR products amplified from the parental genotype being heterozygous for the target SNP. For HRM analysis, a total of twelve mapping individuals along with the parental genotypes were used for short amplicon melting as described by Studer et al. [77]. Primers used for short amplicon melting were designed to flank the target SNP with an amplicon product size of 40 to 60 bp. Sequencing of PCR fragments was performed at Eurofins MWG Operon, Ebersberg, Germany.

### Development of the Lolium oligo pool assay (LOPA1)

LOPA1 used in this study consisted of 786 SNPs selected according to the following criteria: (i) heterozygosity of the target SNP in one or both parental genotypes of VrnA, (ii) absence of additional polymorphisms adjacent to the target SNP, (iii) the detected SNPs were located within a distance of 50 bp to sequence ends or intron/exon splice junctions (iv), absence of polymorphism in sequence reads of the highly inbred reference genotype p226/179/2 within a contig and (v) Illumina assay design score > 0.6 as determined by the Illumina Technical Service. The final set of 768 SNPs addressed 760 ryegrass unigenes, out of which eight were covered with two SNPs.

### SNP genotyping

The parental genotypes of the VrnA mapping population were genotyped in duplicate. Genotyping was performed according to the manufactures protocol on 96-well format Sentrix arrays [98] using the BeadArray technology in combination with an allele-specific extension, adapter ligation and amplification assay protocol. Arrays were imaged using a BeadArray Reader Scanner. Genotyping data generated by the Software Illumina® GenomeStudio, version 2009.2 were manually inspected and corrected for misclassification of genotypes.

### Linkage analysis and map construction

The genetic linkage map of the VrnA population illustrated in Jonavičienė et al. [17] was complemented with 509 gene-associated SNPs. Markers were assigned to LGs using independence LOD scores for group formation. Map construction was based on regression mapping at LOD and recombination threshold value of 1.00 and 0.40, respectively, using the software package JoinMap 4.0 [55]. Map distances were calculated using the Haldane mapping function implemented in JoinMap 4.0.

The annotation of mapped unigenes, including a thorough description of their molecular functions, biological processes and cell compartments involved, was determined based on Gene Ontology (GO) using the Blast2GO search tool [56].

### Heat map construction

The marker density from the ryegrass transcriptome map was visualized by counting the number of markers in a window size of 3 cM shifted in 0.3 cM steps along a linkage group using a manual python script. Color scale was adapted to the minimum (dark blue = 0 marker/3 cM) and maximum (red = 17 to 52 marker/3 cM) window counts, adjusted for each LG separately.

## Competing interests

The authors declare that they have no competing interests.

## Authors’ contributions

BS, TL and TA conceived the study. TA extracted RNA, CB and FP coordinated the sequencing and performed SNP discovery. TA and BS designed LOPA1, MSI and BS validated selected SNPs prior to GoldenGate genotyping. BS coordinated the GoldenGate assay, extracted the mapping data and performed the linkage mapping. BS drafted the manuscript, which was improved by TA, SB, MP and TL. All authors read and approved the final manuscript.

## Supplementary Material

Additional file 1 **Figure S1. SNP validation by high resolution melting (HRM) curve analysis (A) and direct sequencing of PCR fragments (B).** (A) shows the normalized melting curves of a target SNP for twelve mapping individuals along with the parental genotypes that were used for short amplicon melting as described by Studer et al. [77]. The melting curves given in grey represent individuals being homozygous for the target SNP, while red melting curves indicated heterozygous individuals. The sequencing trace file given in (B) illustrates the results from direct sequencing of PCR products amplified from the parental genotype being heterozygous for the target SNP. Sequencing of PCR fragments was performed at Eurofins MWG Operon, Ebersberg, Germany. Click here for file

Additional file 2 **Detailed description of SNP markers.** This table contains the unigene names and GenBank accession numbers along with detailed mapping information (the linkage group and map position) and the SNP polymorphism used for GoldenGate genotyping. Click here for file

Additional file 3 **Figure S2.Heat map of DNA markers on the perennial ryegrass transcriptome map.** Marker density on each linkage group (LG) was visualized as heat maps by counting the number of markers in a window of 3 centi Morgan (cM) size shifted in 0.3 cM steps along a LG using an in-house python script. Color scale was adapted to the minimum (dark blue = 0 marker/2 cM) and maximum (red = 17 to 52 marker/3 cM) window counts, adjusted for each LG separately. Click here for file

Additional file 4 **Figure S3.Summary of unigene annotation.** The 732 non redundant Lolium unigenes were subjected to a BLASTN search against the non-redundant (nr) nucleotide database of Genbank, mapped and functionally annotated based on Gene Ontology (GO) using the Blast2GO search tool [56]. Click here for file

Additional file 5 **Figure S4. Description of biological processes affected by mapped Lolium unigenes.** Biological processes were determined based on Gene Ontology (GO) using the Blast2GO search tool [56]. The number of mapped unigenes involved in a specific process is given in parenthesis. Click here for file

Additional file 6 **Figure S5.Description of cellular components involved in molecular functions of mapped Lolium unigenes.** Mapped unigenes were allocated to cellular components based on Gene Ontology (GO) using the Blast2GO search tool [56]. The number of unigenes for each cellular component is given in parenthesis. Click here for file
